# Generation of Complex Transverse Energy Flow Distributions with Autofocusing Optical Vortex Beams

**DOI:** 10.3390/mi12030297

**Published:** 2021-03-12

**Authors:** Svetlana N. Khonina, Alexey P. Porfirev, Andrey V. Ustinov, Muhammad Ali Butt

**Affiliations:** 1 IPSI RAS—Branch of the FSRC “Crystallography and Photonics” RAS, Molodogvardeyskaya 151, 443001 Samara, Russia; porfirev.alexey@ipsiras.ru (A.P.P.); andr@ipsiras.ru (A.V.U.); 2 Department of Technical Cybernetics, Samara National Research University, MoskovskoyeShosse 34, 443086 Samara, Russia

**Keywords:** optical vortex, autofocusing beams, rotating beams, transverse energy flow density

## Abstract

Optical vortex (OV) beams are widely used for the generation of light fields with transverse energy flow inducing orbital motion of the nano- and microparticles in the transverse plane. Here, we present some new modifications of OV beams with autofocusing properties for shaping complex transverse energy flow distributions varying in space. The angular component of the complex amplitude of these beams is defined by the superpositions of OV beams with different topological charges. The proposed approach provides a convenient method to control the three-dimensional structure of the generated autofocusing OV beams. The control of the transverse distribution of an autofocusing beam provides a wide variety of generated fields with both rotating and periodic properties, which can be used in the field of laser manipulation and laser material processing. Thus, the obtained numerical results predict different types of motion of the trapped particles for the designed OV autofocusing beams. The experimental results agree with modeling results and demonstrate the principal possibility to shape such laser beams using spatial light modulators.

## 1. Introduction

Structured optical beams with given 3D distribution and characteristics are in demand in various applications, information on which can be found in research and review publications [1,2,3,4,5,6,7,8,9,10]. An interesting type of beams with controlled 3D characteristics is those with autofocusing properties. Such beams can autofocus without lenses or nonlinearity in the optical medium. The main characteristics of autofocusing, such as the curvature of the caustic trajectory, focusing sharpness, and focusing distance, are determined by the type of dependence of the input function on the radius. At present, the various types of autofocusing beams are: circular Airy beams [11,12,13,14,15], circular Pearcey beams [16,17,18], aberration beams [19], as well as a mirror [20,21] and generalized [22,23,24,25] Airy beams.

Among the main applications of autofocusing beams are optical trapping and manipulation [26,27,28] and laser material processing [29,30]. In these applications, not only is the character of autofocusing (sharpness and focus position) important, but also the transverse field distribution. For example, the control of the transverse structure of the intensity distribution allows fabricating different nano-microstructures with the desired shape using pulsed laser radiation [31]. The trapped particles move in the direction of the intensity and phase gradient [32]. One of the methods to vary this distribution is to supplement an axisymmetric autofocusing beam with amplitude and phase modulation [13,14,15,18,19,20,23,33,34,35,36], among which the most interesting is the vortex phase since it is preserved during propagation. Inserting a vortex phase singularity into a laser beam can lead to various polarization and spin-orbit transformations [37,38,39,40].

An important characteristic of the generated field for optical trapping problems is the transverse energy flow density (TEFD), which is proportional to the product of the field intensity and phase gradient [41,42]. Inserting a vortex singularity leads to variations in the phase gradient and provides an opportunity to control the TEFD [32].

In this paper, we consider fields with a complex amplitude in the form of the product of the circular Airy function and a combination of optical vortices. The first factor ensures the fact of autofocusing, and optical vortices change the transverse structure of the field. This approach is convenient for experimental implementation since separate optical elements can be used to control the autofocusing properties and the transverse beam structure.

The superposition of optical vortices allows the formation of rotating (spiral) autofocusing beams during propagation, as well as varying the TEFD in the focal area. Autofocusing beams rotating during propagation have attracted the attention of researchers [23,35,36] due to the transverse beam structure varying depending on the distance, which expands the possibilities of using such beams in various applications. Note that in these works, to obtain the rotation effect, either the spatial combination of different beams was used, for example, by sectors [35], rings [36], or inserting a nonlinear vortex phase into a beam [23]. The second approach is more convenient for experimental implementation; however, the nonlinear vortex phase provides the formation of one type of transverse distribution in the form of a spiral [43].

In this paper, we consider the control of the transverse distribution of an autofocusing beam due to the coaxial superposition of several optical vortices, which can provide a wide variety of generated fields with both rotating and periodic properties [44,45]. The results of modeling the distribution of intensity and TEFD and the experimental results of measuring the intensity are in good agreement.

## 2. Theoretical Analysis

Let us consider vortex fields with autofocusing properties in the following form:

(1) $$f(r,\varphi )\;=\;A\left(r\right)\Phi \left(\varphi \right)\;=\;A\left(r\right)\sum_{l=1}^{L}\exp\left(im_{l}\varphi \right)$$

where A(r) is a real function depending only on the radius (for example, circular Airy function, circular Pearcey function, or radial sinus function) and $\exp\left(im_{l}\varphi \right)$ is the optical vortex with the topological charge $m_{l}$.

The main characteristics of autofocusing, such as the curvature of the caustic trajectory and the focusing distance, are determined by the function A(r) in Equation (1). However, in applications such as microparticles trapping and laser structuring, the transverse field distribution in the focal region is also important. As a rule, variations in the transverse distribution are achieved by amplitude and phase modulation of an axisymmetric beam A(r) [13,14,15,18,19,20,23,33,34,35,36].

The representation in Equation (1) is convenient for experimental implementation since it is possible to use separate optical elements to control the autofocusing properties provided by the function A(r) and the transverse structure of the beam due to the superposition of optical vortices provided by the function Φ(φ). Note that variations in Φ(φ) can be performed dynamically using the spatial light modulator (SLM).

Factorization of the field into radial and angular components makes it possible to separately perform a theoretical analysis of the autofocusing properties of the field and its transverse structure. Since autofocusing properties that depend on the radial part of the field A(r) have been investigated in previous works listed, for example, in the Introduction, in this section, we focus on the analysis of the transverse properties associated with the angular part Φ(φ).

Let us consider the paraxial propagation of the beam (1) in free space at a distance *z* using the Kirchhoff–Fresnel integral:

(2) $$E(\rho ,\theta ,z)\;=\;-\frac{ik}{2\pi z}\int_{0}^{2\pi }\int_{0}^{R}f(r,\varphi )\exp\left\{\frac{ik}{2z}\left[\rho^{2}\;+\;r^{2}\;-\;2\rho r\cos(\varphi \;-\;\theta )\right]\right\}rdrd\varphi$$

where *k* = 2π/λ is the wavenumber for laser radiation with a wavelength λ.

If we substitute Equation (1) into Equation (2), then the integration dφ can be performed analytically. For one *l*-term, we get:

(3) $$E_{l}(\rho ,\theta ,z)\;=\;{\left(-i\right)}^{m_{l}+1}\frac{k}{z}\exp(im_{l}\theta )\exp\left(\frac{ik}{2z}\rho^{2}\right)\int_{0}^{R}A(r)J_{m_{l}}\left(\frac{k\rho r}{z}\right)\exp\left(\frac{ik}{2z}r^{2}\right)rdr$$

The full field will be:

(4) $$E(\rho ,\theta ,z)\;=\;\frac{k}{z}\exp\left(\frac{ik}{2z}\rho^{2}\right)\cdot \sum_{l=1}^{L}\left[{\left(-i\right)}^{m_{l}+1}\exp(im_{l}\theta )\int_{0}^{R}A(r)J_{m_{l}}\left(\frac{k\rho r}{z}\right)\exp\left(\frac{ik}{2z}r^{2}\right)rdr\right]$$

Let’s rewrite Equation (4) as follows:

(5) $$E(\rho ,\theta ,z)\;=\;B\left(\rho ,z\right)\cdot \sum_{l=1}^{L}C_{l}\left(\rho ,z\right)\exp\left(im_{l}\theta \right)$$

where $B\left(\rho ,z\right)\;=\;\frac{k}{z}\exp\left(\frac{ik}{2z}\rho^{2}\right)$,

(6) $$C_{l}\left(\rho ,z\right)\;=\;{\left(-i\right)}^{m_{l}+1}\int_{0}^{R}A(r)J_{m_{l}}\left(\frac{k\rho r}{z}\right)\exp\left(\frac{ik}{2z}r^{2}\right)rdr$$

It is seen from Equation (5) that the field defined by Equation (1), when propagating, conserves the same set of vortex functions that were at the input plane (at *z* = 0), but the weights of these functions change depending on the radius of the observation point ρ and the propagation distance *z*.

For a detailed analysis of transverse distribution, let us consider the field defined by Equation (5) in some fixed plane $z\;=\;z_{0}$:

(7) $$E\left(\rho ,\theta ,z_{0}\right)\;=\;B\left(\rho ,z_{0}\right)\cdot \sum_{l=1}^{L}C_{l}\left(\rho ,z_{0}\right)\exp\left(im_{l}\theta \right)$$

The TEFD in the plane $z\;=\;z_{0}$ is proportional to the product of the field intensity and phase gradient [32,42,43]:

(8) $$F\left(\rho ,\theta ,z_{0}\right)∼{\left|E\left(\rho ,\theta ,z_{0}\right)\right|}^{2}\nabla \psi \left(\rho ,\theta ,z_{0}\right)$$

where $\psi \left(\rho ,\theta ,z_{0}\right)\;=\;\arg\left[E\left(\rho ,\theta ,z_{0}\right)\right]$ is the phase of the field in the plane $z\;=\;z_{0}$.

The field intensity defined from Equation (7) has the following form:

(9) $${\left|E\left(\rho ,\theta ,z_{0}\right)\right|}^{2}\;=\;{\left(\frac{k}{z_{0}}\right)}^{2}\left\{{\left[\sum_{l=1}^{L}D_{l}\left(\rho \right)\cos\left[m_{l}\theta +\psi_{l}\left(\rho \right)\right]\right]}^{2}+{\left[\sum_{l=1}^{L}D_{l}\left(\rho \right)\sin\left[m_{l}\theta +\psi_{l}\left(\rho \right)\right]\right]}^{2}\right\}$$

where $D_{l}\left(\rho \right)\;=\;\left|C_{l}\left(\rho ,z_{0}\right)\right|$ and $\psi_{l}\left(\rho \right)\;=\;\arg\left[C_{l}\left(\rho ,z_{0}\right)\right]$.

Since we are interested in the radial and angular energy flow directions, we consider the phase gradient in polar projections:

(10) $$\nabla \psi \left(\rho ,\theta ,z_{0}\right)\;=\;\frac{\partial \psi \left(\rho ,\theta ,z_{0}\right)}{\partial \rho }e_{\rho }\;+\;\frac{\partial \psi \left(\rho ,\theta ,z_{0}\right)}{\partial \theta }e_{\theta }$$

The TEFD in the radial and angular directions is of particular importance for applications such as optical trapping and manipulation. The radial energy flow, provided by the autofocusing properties, directs the trapped particles to the optical axis [46], and the angular energy flow associated with the vortex structure of the phase leads to the rotation of the particles [47].

The field distribution in the autofocusing plane $z\;=\;z_{foc}$ near the optical axis is of particular interest since most of the energy is concentrated there. In this case, the angular component TEFD corresponding to the phase derivative concerning the angle in Equation (10) becomes significant. Let us consider it separately. In general, this expression is rather cumbersome:

(11) $$\begin{array}{l} \frac{\partial \psi \left(\rho ,\theta ,z_{0}\right)}{\partial \theta }\;=\;\frac{1}{{\left|E\left(\rho ,\theta ,z_{0}\right)\right|}^{2}}\;\times \\ \times \;\{\left[\sum_{l=1}^{L}D_{l}\left(\rho \right)m_{l}\cos\left[m_{l}\theta \;+\;\psi_{l}\left(\rho \right)\right]\right]\left[\sum_{l=1}^{L}D_{l}\left(\rho \right)\cos\left[m_{l}\theta \;+\;\psi_{l}\left(\rho \right)\right]\right]\;+\\ +\;\left[\sum_{l=1}^{L}D_{l}\left(\rho \right)m_{l}\sin\left[m_{l}\theta \;+\;\psi_{l}\left(\rho \right)\right]\right]\left[\sum_{l=1}^{L}D_{l}\left(\rho \right)\sin\left[m_{l}\theta \;+\;\psi_{l}\left(\rho \right)\right]\right]\} \end{array}$$

Special cases are discussed in detail in the next section.

## 3. Results of Modeling and Experiment

We consider the circular Airy functions as functions depending on the radius:

(12) $$A\left(r\right)\;=\;\mathrm{Ai}\left(\frac{r_{0}\;-\;r}{w}\right)\mathrm{circ}\left(\frac{r}{R}\right)$$

where Ai(*x*) is the Airy function [48], circ(*r*/*R*) is the circle function with unit amplitude, radius *R*, $r_{0}$ is the radial displacement parameter, and *w* is the normalizing parameter.

Instead of function in Equation (12), one can use other circular beams with autofocusing properties, for example, circular Pearcey beams [17,18], chirped beams [22,25,49], and generalized Airy beams [24]. This mainly affects autofocusing characteristics such as focal trajectory curvature, sharpness and focusing distance.

The following parameters were used in the simulation: wavelength λ = 0.532 μm, *w* = 5 mm, $r_{0}$ = 2 mm, and *R* = 1 mm.

**Figure 1:**
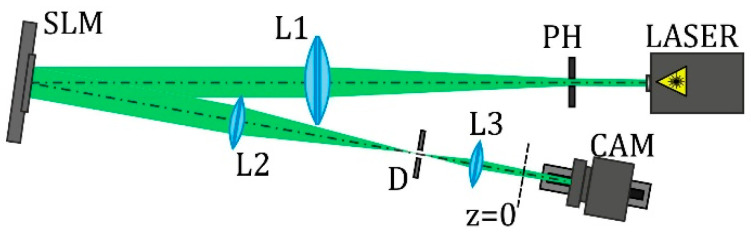
Experimental setup for the investigation of the generation and propagation of designed autofocusing optical vortex (OV) beams: PH, pinhole (aperture size of 40 µm); L1, L2, and L3 are lenses (*f*_1_ = 350, *f*_2_ = 350, and *f*_3_ = 150 mm, respectively); SLM, spatial light modulator (HOLOEYE, PLUTO VIS with a 1920 × 1080 pixel resolution); D, circular aperture; and CAM, video camera.

The experimental parameters coincide with the mentioned simulation parameters. The used experimental setup is shown in Figure 1. The initial linearly polarized laser beam (λ = 532 nm) was extended and spatially filtered by a system composed of a pinhole (PH) (aperture size of 40 µm) and lens L1 (focal length of 350 mm). The collimated laser beam was directed onto a display of a reflective SLM (HOLOEYE, PLUTO VIS with a 1920 × 1080 pixel resolution), which was used to realize the phase masks of the designed elements for the generation of autofocusing optical vortex (OV) beams. Then, the laser beam modulated by the SLM was spatially filtered with a 4f imaging optical system consisting of lenses L2 and L3 (focal lengths of 350 and 150 mm, respectively) and a circular aperture D. This system allowed blocking a portion of laser radiation that was not modulated by SLM due to its pixilated structure. A video camera (CAM) mounted on the optical rail was used to record the generated intensity distributions at different distances from the plane *z* = 0. The longitudinal intensity distributions were reconstructed from arrays of cross-sections of the recorded transverse intensity distributions along the vertical axis.

**Figure 2:**
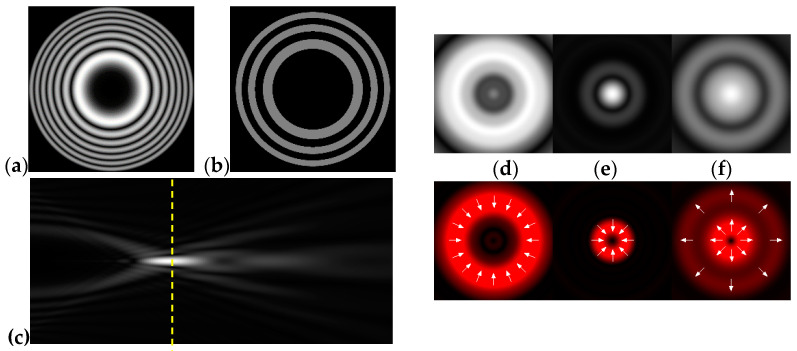
Simulation results for the field defined by Equation (1), which does not have any angular dependence: (**a**) amplitude and (**b**) phase of the input field (x,y ∈ [−1 mm, 1 mm]); (**c**) longitudinal distribution of the amplitude (z ∈ [20 mm, 500 mm], y ∈ [−1 mm, 1 mm]), and transverse patterns (x,y ∈ [−0.25 mm, 0.25 mm]) of the field amplitude at different distances (top line): (**d**) z = 150 mm, (**e**) $z_{foc}\;=\;200\;\mathrm{mm}$, and (**f**) *z* = 250 mm, as well as the corresponding distributions of the transverse energy flow density (TEFD) amplitude in these planes (bottom line). The red color corresponds to the radial component $\left|F_{\rho }\left(\rho ,\theta \right)\right|$, the blue color corresponds to the angular component $\left|F_{\theta }\left(\rho ,\theta \right)\right|$, and the arrows show the direction of flow.

Figure 2 shows the results of propagation modeling for the field defined by Equation (1) with the radial function defined by Equation (13), which does not have any angular dependence. The longitudinal distribution pattern shows that autofocusing occurs at the distance $z_{foc}\;=\;200\;\mathrm{mm}$ (marked with the dashed line). Figure 2 also shows the transverse patterns of the field amplitude at different distances, as well as the corresponding distributions of the TEFD in these planes.

In the considered case, the TEFD only had the radial component. At distances $z\;<\;z_{foc}$, the flow was directed to the optical axis (Figure 2d,e), and after focusing, i.e., at distances $z\;>\;z_{foc}$, the flow was directed away from the optical axis (Figure 2f).

### 3.1. Circular Airy Beams with Vortex Superposition

In this section, we consider the field in Equation (1) with the radial function A(r) as the circular Airy function (12) and the angular function as a vortex superposition $\Phi \left(\varphi \right)=\sum_{l=1}^{L}\exp\left(im_{l}\varphi \right)$. A particular case of a beam with a single vortex phase is $\Phi \left(\varphi \right)=\exp\left(im_{1}\varphi \right)$. In this case, the angular component of the TEFD defined by Equation (11) has the following form:

(13) $$F_{\theta }\left(\rho ,\theta ,z_{0}\right)∼{\left|E\left(\rho ,\theta ,z_{0}\right)\right|}^{2}\frac{\partial \psi \left(\rho ,\theta ,z_{0}\right)}{\partial \theta }\;=\;{\left|C_{1}\left(\rho ,z_{0}\right)\right|}^{2}m_{1}$$

Thus, the speed of rotation of the trapped particle will be proportional to the order $m_{1}$ of the vortex singularity present in the beam.

**Figure 3:**
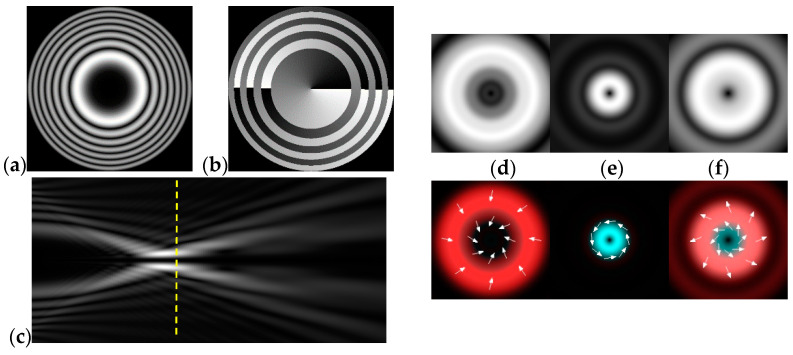
Simulation results for the field defined by Equation (1) in the presence of a single vortex phase of order $m_{1}\;=\;1$ (the rest of the description is as in Figure 2).

Figure 3 shows the results of propagation modeling for the field defined by Equation (1) in the presence of a single vortex phase of the order $m_{1}$. In this case, the TEFD, in addition to the radial component, had an angular component (see Equation (13)). Note that in the autofocusing plane $z_{foc}\;=\;200\;\mathrm{mm}$, the radial component was close to zero, so the main energy flow was directed along the ring (Figure 3e). There were both radial and angular components before and after the focal plane, so the energy flow was spiraled (Figure 3f).

Next, we considered the field containing two vortex terms:

(14) $$f(r,\varphi )\;=\;A\left(r\right)\left[\exp\left(im_{1}\varphi \right)\;+\;\exp\left(im_{2}\varphi \right)\right]$$

In this case, the field intensity in a certain plane $z\;=\;z_{0}$ was:

(15) $$\begin{array}{l} {\left|E\left(\rho ,\theta ,z_{0}\right)\right|}^{2}\;=\;{\left(\frac{k}{z_{0}}\right)}^{2}\{{\left|C_{1}\left(\rho ,z_{0}\right)\right|}^{2}\;+\;{\left|C_{2}\left(\rho ,z_{0}\right)\right|}^{2}\;+\\ +\;2\left|C_{1}\left(\rho ,z_{0}\right)\right|\left|C_{2}\left(\rho ,z_{0}\right)\right|\cos\left[\left(m_{1}\;-\;m_{2}\right)\theta \;+\;\arg\left[C_{1}\left(\rho ,z_{0}\right)\right]\;-\;\arg\left[C_{1}\left(\rho ,z_{0}\right)\right]\right]\} \end{array}$$

As follows from Equation (15), the angular structure of the field depend only on the difference in the orders of optical vortices $\left(m_{1}\;-\;m_{2}\right)$, whereas variations in the radial functions $C_{l}\left(\rho ,z_{0}\right)$ lead only to a scale change and rotation of this structure. A similar result was obtained in [44,45].

The phase is calculated based on the expression:

(16) $$\tan\left[\psi \left(\rho ,\theta ,z_{0}\right)\right]\;=\;\frac{D_{1}\left(\rho \right)\sin\left[m_{1}\theta \;+\;\psi_{1}\left(\rho \right)\right]\;+\;D_{2}\left(\rho \right)\sin\left[m_{2}\theta \;+\;\psi_{2}\left(\rho \right)\right]}{D_{1}\left(\rho \right)\cos\left[m_{1}\theta \;+\;\psi_{1}\left(\rho \right)\right]\;+\;D_{2}\left(\rho \right)\cos\left[m_{2}\theta \;+\;\psi_{2}\left(\rho \right)\right]}$$

Since $D_{1}\left(\rho \right)\;\neq \;D_{2}\left(\rho \right)$, the form of the formula for the phase gradient defined by Equation (11) and intensity defined by Equation (9) was not simplified compared with the general case *L* > 2.

Table 1 shows the results of simulations and experiments for the field defined by Equation (14) with different orders of vortex terms $m_{1},\;m_{2}$.

The asymmetry concerning sign change $m_{2}$ is easily explained based on Equation (15). When using a superposition of two vortex fields, the symmetry of the generated field is determined by the difference $\left|m_{1}\;-\;m_{2}\right|$. In the examples considered, $m_{1}\;=\;2,\;m_{2}\;=\;1$, the input field amplitude (first column of Table 1) should be equal to zero at φ = 180°, and for $m_{1}\;=\;2,\;m_{2}\;=\;-1$, at φ = 60°, 180°, 300°. The transverse structure was preserved during propagation with an accuracy of scale and rotation, which is fully confirmed by both the simulation and experiment.

The corresponding distributions of the TEFD show that particles trapped in the region of maximum intensity will rotate spirally as a whole structure, decreasing on a scale when moving to the focal plane and expanding after it. The number of maxima and the symmetry of the transverse pattern are entirely determined by the function Φ(φ), which can be dynamically varied using SLM.

The use of more than three vortex terms in Equation (1) will complicate the formed patterns and increase the number of degrees of freedom in variations of the transverse field pattern [50]; however, two terms are sufficient to form a wide variety of structures [51].

The proposed approach based on the representation of the angular part of the field defined by Equation (1) in the form of a superposition of optical vortices provides a simple and convenient method to control the 3D structure of autofocusing beams.

### 3.2. Azimuthally Modulated Circular Vortex Airy Beams

In [14], azimuthally modulated circular Airy beams were investigated, which can be considered a special case of the field defined by Equation (14), with $m_{2}\;=\;-m_{1}\;=\;q$ (*q* is an arbitrary integer):

(17) f(r,φ) = A(r)cos(qφ)

In this case, dψ/dθ = 0 (except for the lines of phase jumps), since the phase is piecewise constant (has values 0 and π).

**Figure 4:**
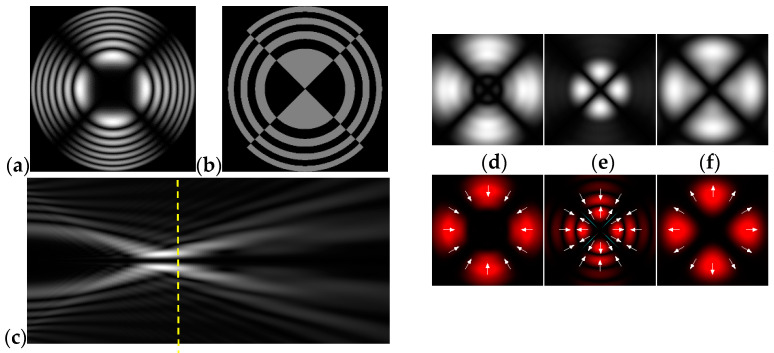
Simulation results for the field defined by Equation (17) with azimuthal modulation of the order of *q* = 2 (the rest of the description is as in Figure 2).

Figure 4 shows the simulation results for the field defined by Equation (17) with azimuthal modulation of the order of *q* = 2. The TEFD only has a radial component: before focusing ($z\;<\;z_{foc}$), the energy flow is directed toward the optical axis (Figure 4d), and after focusing ($z\;>\;z_{foc}$), the energy flow is directed from the optical axis (Figure 4f). The difference compared with classical circular autofocusing beams is that the bright region is separated by zero-intensity stripes in angular sectors, and the motion of trapped particles is limited not only in the radial but also in the angular direction. In the autofocusing plane ($z\;=\;z_{foc}$) the energy flow has the opposite direction on the adjacent rings (Figure 4e), which corresponds to its absence in the transverse direction. Note that these rings were not observed in the field amplitude picture, since they arose due to the phase gradient.

Let us consider the beam defined by Equation (17) with an additional vortex phase singularity:

(18) f(r,φ) = A(r)cos(qφ)exp(imφ)

The field defined by Equation (18) can be represented as the sum of two vortex terms:

(19) $$f(r,\varphi )\;=\;A\left(r\right)\left[\exp\left(ip_{1}\varphi \right)\;+\;\exp\left(ip_{2}\varphi \right)\right]$$

where $p_{1}\;=\;q\;+\;m$, $p_{2}\;=\;q\;-\;m$.

Equation (19) coincides with Equation (14) up to notation. However, the situation considered in this section differs from that considered in the previous section. We initially had a beam structure divided into angular sectors (azimuthally modulated autofocusing beam) and varied it due to the additionally inserted vortex phase.

Equation (19) shows that for *m* = ±*q*, one of the terms becomes a constant, which provide a nonzero value of the intensity on the optical axis in the autofocusing region. This is another difference from the situation discussed in the previous section.

Table 2 shows the results of the simulation and experiment for the field defined by Equation (18) with azimuthal modulation of the order of *q* = 2 and with different orders of additional vortex phase singularity *m*.

It follows from Equation (18) that at *q* = 2, regardless of *m*, the amplitude of the input field should be equal to zero φ = 45°, 135°, 225°, 315°. The *m* value only affects the phase structure. Both dependencies are fully confirmed by the pictures in the first column.

We argue that the quantity *q* determines the order of symmetry of the transverse intensity distribution, which is equal to 2*q*, while the detailed picture of the distribution, including the value of the field at the optical axis in the focal region, depends on the quantity *m*. When *m* = ±*q*, the central spot was at the optical axis.

Detailed results of the TEFD investigation in the focal region for the considered beams are shown in Table 3. The effects of the additional vortex phase singularity are most significant in the focal plane but are also important in the region after autofocusing. This can be explained by the interference interaction of different parts of the beam, which is not present before the focal region. In the areas before and after the focus, the spiral nature of the energy flow is visible. In the plane of focusing, one can observe a more complex structure associated with the influence of an additional vortex, including the presence of several regions with different directions of the angular motion of the energy flow.

As seen from the results in Table 2 and Table 3, inserting an additional vortex phase singularity into the beam defined by Equation (17) significantly changes the field distribution in the autofocusing region.

Comparison of the results of Section 3.1 and Section 3.2 (Table 1 and Table 3) shows that using the proposed approach, different scenarios for controlling the transverse intensity structure and TEFD of autofocusing beams are possible. In the first case, a set of bright spots can be formed in a cross-section, which rotates as a single structure when the longitudinal distance changes. In the second case, a certain symmetry of the transverse structure is fixed, and the inserting of an additional vortex phase allows it to be changed locally, ensuring the presence or absence of a central light spot. The latter property is useful in the formation of optical bottles based on autofocusing beams.

## 4. Conclusions

In this paper, we considered the possibility of controlling the transverse intensity structure and TEFD of autofocusing beams by inserting a combination of optical vortices into the beam. This approach is convenient for experimental implementation since a separate optical element can be used to form an autofocusing beam, while variations in the transverse distribution can be performed dynamically using SLM.

Based on the proposed approach, we showed, numerically and experimentally, the possibility of forming autofocusing beams, the transverse intensity pattern of which rotates as a whole structure during beam propagation. The corresponding distributions of the TEFD show that particles trapped in the light spots rotate spirally as a single structure, decreasing on a scale when moving to the focal plane and expanding after it. The structure and symmetry of the transverse pattern are determined by the angular function, which is a superposition of optical vortices.

Another option is considered when an azimuthally modulated autofocusing beam with an additional vortex phase is used. In this case, a certain symmetry of the transverse structure is fixed, and the inserting of the vortex phase allows it to be changed locally, ensuring the presence or absence of a central light spot, which can be useful in the formation of optical bottles. The effect of the additional vortex phase is most significant near the focusing region. Complex distributions of the TEFD are formed, including those with several areas with different directions of angular energy flow.

This variety of configurations is a useful property in the implementation of the trapping/confinement of particles, especially when using dynamic optical elements, in particular SLM. For example, a sufficiently large annular region is convenient for initial trapping, after which the element switches to creating sector regions that narrow the particle localization area. The proposed approach provides a simple and convenient way to control the 3D structure of autofocusing beams.

## Figures and Tables

**Table 1:** Results of simulations and experiments for the field defined by Equation (14) with different orders of vortex terms *m*_1_, *m*_2_.

Input Amplitude and Phase (2 mm × 2 mm)	Longitudinal Intensity Distribution (2 mm × 300 mm)	Transverse Distribution		
		*z* = 150 mm	*z* = 200 mm	*z* = 250 mm
*m*_1_ = 2, *m*_2_ = 1 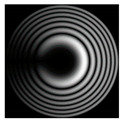 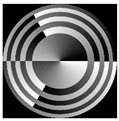	Simulation 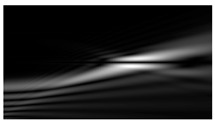 Experiment 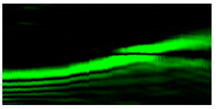	Simulation: intensity (1.6 mm × 1.6 mm) 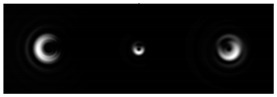 Simulation: TEFD (1 mm × 1 mm) 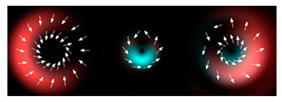 Experiment: intensity (1.6 mm × 1.6 mm) 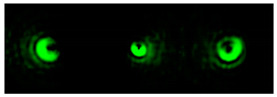		
*m*_1_ = 2, *m*_2_ = −1 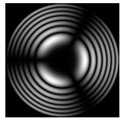 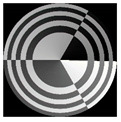	Simulation 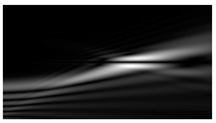 Experiment 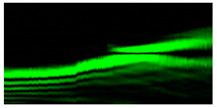	Simulation: intensity (1.6 mm × 1.6 mm) 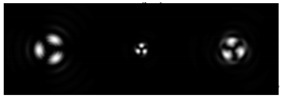 Simulation: TEFD (1 mm × 1 mm) 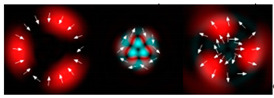 Experiment: intensity (1.6 mm × 1.6 mm) 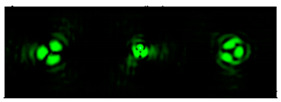		

**Table 2:** Results of modeling and experiment for the field defined by Equation (18) with azimuthal modulation of order *q* = 2 with different orders of additional vortex phase singularity *m*.

Input Amplitude and Phase (2 mm × 2 mm)	Longitudinal Intensity Distribution (1.6 mm × 300 mm)	Transverse Intensity Distribution (1.6 mm × 1.6 mm)		
		***z* = 100 mm**	***z* = 200 mm**	***z* = 300 mm**
*q* = 2, *m* = 1	Simulation	Simulation		
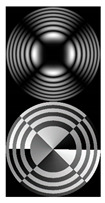	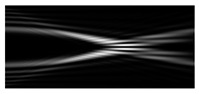	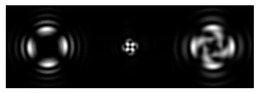		
	Experiment	Experiment		
	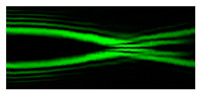	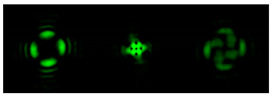		
*q* = 2, *m* = 2	Simulation	Simulation		
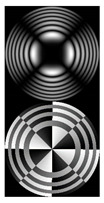	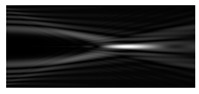	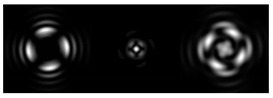		
	Experiment	Experiment		
	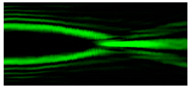	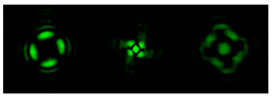		

**Table 3:** Results of TEFD simulation for the field defined by Equation (18) with azimuthal modulation of order *q* = 2 and with different orders of additional vortex phase singularity *m*.

Input Amplitude and Phase (2 mm × 2 mm)	**Transverse Distribution (1 mm × 1 mm)**	
	Value	*z* = 150 mm *z* = 175 mm *z* = 200 mm *z* = 225 mm *z* = 250 mm
*q* = 2, *m* = 1 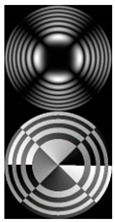	|E(ρ,θ)|	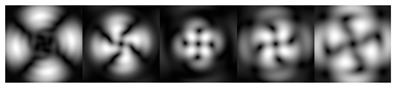
	|F(ρ,θ)|	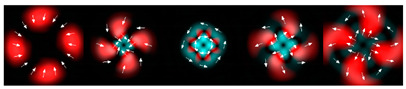
*q* = 2, *m* = 2 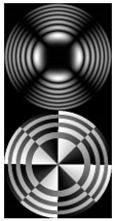	|E(ρ,θ)|	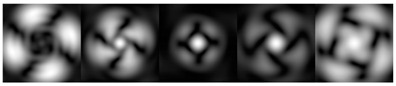
	|F(ρ,θ)|	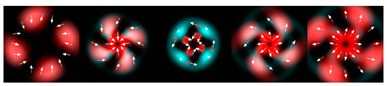

## Data Availability

Not applicable.
